# Cross-Frequency Coupling Based Neuromodulation for Treating Neurological Disorders

**DOI:** 10.3389/fnins.2019.00125

**Published:** 2019-02-21

**Authors:** Yousef Salimpour, William S. Anderson

**Affiliations:** Functional Neurosurgery Laboratory, Department of Neurosurgery, Johns Hopkins School of Medicine, Baltimore, MD, United States

**Keywords:** cross-frequency coupling, phase-amplitude coupling, electrocorticography, center out reaching task, sequence learning, memory, Parkinson’s disease, epilepsy

## Abstract

Synchronous, rhythmic changes in the membrane polarization of neurons form oscillations in local field potentials. It is hypothesized that high-frequency brain oscillations reflect local cortical information processing, and low-frequency brain oscillations project information flow across larger cortical networks. This provides complex forms of information transmission due to interactions between oscillations at different frequency bands, which can be rendered with cross-frequency coupling (CFC) metrics. Phase-amplitude coupling (PAC) is one of the most common representations of the CFC. PAC reflects the coupling of the phase of oscillations in a specific frequency band to the amplitude of oscillations in another frequency band. In a normal brain, PAC accompanies multi-item working memory in the hippocampus, and changes in PAC have been associated with diseases such as schizophrenia, obsessive-compulsive disorder (OCD), Alzheimer disease (AD), epilepsy, and Parkinson’s disease (PD). The purpose of this article is to explore CFC across the central nervous system and demonstrate its correlation to neurological disorders. Results from previously published studies are reviewed to explore the significant role of CFC in large neuronal network communication and its abnormal behavior in neurological disease. Specifically, the association of effective treatment in PD such as dopaminergic medication and deep brain stimulation with PAC changes is described. Lastly, CFC analysis of the electrocorticographic (ECoG) signals recorded from the motor cortex of a Parkinson’s disease patient and the parahippocampal gyrus of an epilepsy patient are demonstrated. This information taken together illuminates possible roles of CFC in the nervous system and its potential as a therapeutic target in disease states. This will require new neural interface technologies such as phase-dependent stimulation triggered by PAC changes, for the accurate recording, monitoring, and modulation of the CFC signal.

## Introduction

Neuronal rhythmic activities are observed across various temporal and spatial scales in both cortical areas and deep structures (Canolty and Knight, 2010) in healthy subjects and neurological disorders (Wang, 2010; Hyafil et al., 2015) including: schizophrenia (Allen et al., 2011), obsessive-compulsive disorder (OCD) (Bahramisharif et al., 2016), Alzheimer’s disease (Goutagny et al., 2013), and Parkinson’s disease (De Hemptinne et al., 2013) and recently in epilepsy (Zhang et al., 2017). These brain oscillations have specific structures and may play a crucial role in synchronization of neuronal spiking activity used during processing of sensory inputs across wide cortical network such as visual object perception (Engel et al., 1991a,b, 1992). One of the unique features of neural oscillations is that rhythms of distinct frequencies show specific coupling properties, such as cross-frequency coupling (CFC) which potentially provides a mechanism for synchronization and interaction between local and global processes across wide cortical networks (Florin and Baillet, 2015). In general, CFC is mostly categorized as: amplitude–amplitude coupling, phase-phase coupling, phase-amplitude coupling (PAC) and phase–frequency coupling (Jirsa and Müller, 2013). Among existing studies, the most comprehensive example of CFC is between theta and gamma oscillatory activity in the memory system of both humans and animals (Canolty and Knight, 2010; Lisman and Jensen, 2013).

Investigations of CFC have been performed primarily in memory studies. Electrocorticographic (ECoG) recordings from brain regions active in memory function (i.e., inferior and medial aspects of the temporal lobe within the parahippocampal gyrus and in the hippocampus) in human subjects and of different animal species provide evidence that sustained increases of gamma activity (Gruber et al., 2002; Howard, 2003; Axmacher et al., 2007) and theta oscillations (Raghavachari et al., 2001; Summerfield and Mangels, 2005) are a neural correlate of memory related tasks such as working memory maintenance. Some studies show more sophisticated mechanisms including the coupling between the spiking activity of single neurons to the specific phases of ongoing theta oscillations (O’Keefe and Recce, 1993; Rutishauser et al., 2010) and also firing rate modulation by the power and phase of gamma and high gamma rhythms (Csicsvari et al., 2003; Ray et al., 2008; Senior et al., 2008).

Additionally, more evidence has emerged to support the role of this form of coupling in the maintenance of multiple items in working memory (Jensen and Lisman, 2005; Jensen and Colgin, 2007). Specifically, interactions between the phase of the theta rhythms and gamma power oscillations during task performance both in the human brain (Palva et al., 2005; Canolty et al., 2006; Axmacher et al., 2010) and in rodents (Tort et al., 2008) has been demonstrated. CFC is not restricted to memory processes, and has been reported in sensory processing, including vision and visual attention (Seymour et al., 2017; Pascucci et al., 2018; Spyropoulos et al., 2018), olfaction (Pena et al., 2017), and auditory perception (Malinowska and Boatman-Reich, 2016; Kikuchi et al., 2017). A recent observation from nociception processing demonstrated that coupling between theta and gamma is involved during nociception in the rat (Wang et al., 2011). It also can be induced by general anesthetic agents (Li et al., 2012).

We believe there is a scientific, as well as clinical, basis for investigating the role that CFC has in healthy subjects and in patients with neurological disorders. Among different types of CFC, PAC has attracted attention in studies of neurological disorders. It has been shown that different neurological diseases can alter PAC characteristics (Allen et al., 2011; De Hemptinne et al., 2013; Goutagny et al., 2013; Bahramisharif et al., 2016; Widge et al., 2016; Zhang et al., 2017; Hirano et al., 2018) for related rhythmic activity. This increases the potential applications of CFC detection and modulation for possible diagnosis or treatment of nervous system disease. To this end, and as illustrative examples, we demonstrate the CFC analyses of ECoG signals recorded from the motor cortex of a Parkinson’s disease patient and from the parahippocampal gyrus of an epilepsy patient. Additionally, we used our previously developed phase-dependent stimulation algorithm (Chen et al., 2011, 2013) to demonstrate the potential capability of phase-dependent stimulation on changing the CFC signal. This information may illuminate the possible roles of CFC in the nervous system’s normal function and possible correlations with neurological disorders and potential therapeutic targets in disease states.

## Neural Mechanisms of CFC

Despite the fact that CFC has received enormous attention in recent studies in both healthy subjects and neurological disorders, the neurophysiological relevance for CFC has not been fully understood and is still under investigation. There are some hypotheses that link current computational models to the recorded data in human and animal studies and emphasize that computational models could potentially clarify the neurobiological mechanism underlying CFC generation in parallel to experimental studies (Korotkova et al., 2011; Hyafil et al., 2015; Chehelcheraghi et al., 2016, 2017). The results of a modeling study demonstrated how a computational model which included pyramidal cells, fast-spiking basket cells, and oriens-lacunosum moleculare interneurons can reproduce theta–gamma modulation (Neymotin et al., 2011a,b). Additionally, a number of fundamental properties are required for a brain network to generate CFC such as neural oscillations at multiple distinct frequencies, and also different coupling mechanism between the individual neuronal circuits responsible for rhythmic activities. The former property relies on the synaptic coupling between excitatory and inhibitory populations and also, the electrical coupling between individual neurons through gap junctions (Hyafil et al., 2015). The latter feature is considered a main factor for generating different forms of CFC coupling such as amplitude–amplitude coupling (AAC), phase-phase coupling (PPC), phase-amplitude coupling (PAC), and phase–frequency coupling (Jirsa and Müller, 2013; Hyafil et al., 2015).

Phase-amplitude coupling is a commonly used form of CFC and represents the coupling between the phase of a low frequency rhythm (beta or theta range) and amplitude of gamma oscillations. It has been reported in various behavioral experiments in healthy conditions and disease states (Canolty and Knight, 2010; van der Meij et al., 2012). Gamma activities may arise from interactions within populations of highly interconnected inhibitory neurons, as well as from network interactions between populations of excitatory local interneuron networks (Cardin et al., 2009). Theta activity is the result of interneuronal network activity that involves both pyramidal cells and interneurons and their interactions in generating a range of oscillations in the theta band (Bao and Wu, 2003; Whittington and Traub, 2003). Based on recent observations, neuronal population activities are modulated in specific temporal patterns that are delineated by theta oscillations phases (Haider et al., 2006; Lisman and Buzsaki, 2008), and induce the occurrence of neural spiking and faster post-synaptic activity, manifested by gamma-frequency and broadband bursts (Canolty and Knight, 2010). Importantly, it has recently been shown that theta–gamma interactions depend on a particular class of GABAergic interneurons and this is supported by similar behavior in a computational model of the hippocampus (Wulff et al., 2009).

The effects of neurotransmitter release (such as dopamine) on CFC is a relevant question for understanding the neurophysiology of neuronal coupling. In a recent study, the effects of dopamine release on CFC modulation between different frequency bands was explored (Andino-Pavlovsky et al., 2017). PAC between the phase of the theta rhythm and the amplitude of gamma oscillations in the medial prefrontal cortex (mPFC) of behaving rats was analyzed. This work demonstrated a dynamical PAC transition for gamma amplitude coupling from the theta phase to the delta phase using a dopamine uncaging process via light activation. Despite the small sample size limitations, the results suggested that the shift in phase coupling from theta to delta recorded after caged-dopamine (with two-photon sensitivity) uncaging can be related to dopamine interaction with dopamine receptors in the mPFC (Andino-Pavlovsky et al., 2017).

## CFC Modulation in Behavioral Tasks

Robust CFC between theta and gamma oscillations, wherein the amplitude of the gamma band is modulated by the phase of the theta rhythm, seems to play a role in coordinating neuronal spiking throughout widely dispersed cortical networks (Canolty and Knight, 2010; Lisman and Jensen, 2013). Yet, understanding of the mechanisms aided by this CFC in parallel or serial processing is still relatively incomplete. Recent research based on functional connectivity models have found that there is correlational PAC of hippocampal (spatial memory) theta and prefrontal (working memory) gamma bands in rats during the decision point of a T-maze task and in item-context associations, providing evidence for task-dependent cross-frequency modulation in the integration of varied types of information from disparate brain regions (Tort et al., 2008, 2009). Similar ECoG results have been obtained from human subjects via subdural electrode implantation, providing strong indicators that the power of gamma band activity is phase-locked to theta oscillations in the neocortex during different behavioral tasks (Canolty et al., 2006). Several other studies have reported CFC recording results in different brain regions in a variety of experimental conditions correlated with distinct cognitive processes (Axmacher et al., 2010; Fell and Axmacher, 2011; Jensen et al., 2012, 2014; Giraud, 2013; Lisman and Jensen, 2013). Among these observation, one recent study showed a strong correlation between behavioral performance and CFC strength in a learning task (Tort et al., 2009). During this learning task, the strength of CFC in hippocampal regions increased over time with respect to the improvement of the performance, and interestingly both reached a plateau almost at the same time. In fact, this learning specific correlation implies that PAC (as a common form of CFC) constitutes a potential neurophysiological marker for predicting learning and memory behavior (Tort et al., 2009).

## CFC Modulation in Neurological Disorders

Determining correlations between theta–gamma CFC and observable behavioral alterations including cognitive or memory functional changes also has immense potential clinical value. However, whether deterioration or exaggeration of high or low CFC produces disease-specific improvements remains a relatively unanswered question. Studies implicate exaggerated cortical CFC recorded from motor cortex, associated with the movement deficits in Parkinson’s disease (De Hemptinne et al., 2013). However, in schizophrenia, CFC appears to be comparable to healthy subjects, but several components of theta and gamma oscillations appear to be abnormal in the temporal lobe, including the auditory cortex (Moran and Hong, 2011; Kirihara et al., 2012). Especially important for memory is the finding that Alzheimer’s disease may be associated with disruptions of intra-hippocampal gamma-theta CFC (Goutagny et al., 2013). Exploring the role gamma-theta CFC plays in visual memory learning also holds potential application for medical treatments targeting impairments in cognitive functioning found in patients with epilepsy in dorsal and ventral visual cortical regions (Voytek et al., 2010).

Among the neurological disorders, Parkinson’s disease and its correlation to CFC and particularly in PAC, is a well-studied example of the role of CFC in disease pathophysiology and its change over the course of effective treatments (De Hemptinne et al., 2013, 2015; Devergnas et al., 2017). The association between abnormal PAC in motor cortex and Parkinson’s disease has recently being investigated in PD patients undergoing DBS lead placement surgery. The results demonstrated abnormal excessive coupling between the phase of beta band rhythms and the amplitude of the gamma band activities in comparison with controls (De Hemptinne et al., 2013). This is one of the earliest pieces of evidence indicating the strong association between PAC and PD using primary motor cortex local field potential recordings as a biomarker of disease. More importantly, strong correlations between the severity of disease symptoms and level of PAC have been shown along with significant reductions in PAC reported in line with reduction of motor symptoms as a result of DBS of the basal ganglia. Supporting this finding, cortical PAC changes in a drug-induced progressive model of PD in non-human primates was explored and the effects of levodopa treatment on normalization of the coupling level were investigated. The results similarly showed that PD is associated with increased levels of PAC which are proportional to the severity of motor symptoms. Levodopa treatment reduced the Parkinson’s symptoms and normalized the increased PAC at the same time (Devergnas et al., 2017).

A recent study on exploring CFC in neuropsychiatric disorders reported abnormal PAC in OCD (Bahramisharif et al., 2016). ECoG data recorded from the visual cortex of OCD patients contained exaggerated PAC between the phase of the beta band frequency and gamma band oscillations, similar to PAC reported for the motor cortex of PD patients. Additionally, modulation of the striatal activity through DBS in the ventral internal capsule normalized the pattern of PAC and reduced the disease-specific abnormality of CFC within the brain in OCD patients (Bahramisharif et al., 2016). Although this result supports deep brain stimulation (DBS) as a potential treatment for OCD, the results are mixed and the consistency of the outcomes are still under debate, implying the need for a more comprehensive study (Widge et al., 2016).

## Phase-Dependent Stimulation

The idea of phase-dependent stimulation was initially described in studies of long-term potentiation (LTP) and long-term depression (LTD) in the hippocampus (Pavlides et al., 1988; Otto et al., 1991; Stewart et al., 1992; Huerta and Lisman, 1993, 1995). When stimuli were locked to a specific phase of the low-frequency theta oscillation an induced modulatory effect on LTP and LTD has been observed both *in vivo* and *in vitro* (Pavlides et al., 1988; Huerta and Lisman, 1993; Jeffery et al., 1996). Specifically, homosynaptic LTP induction was achieved when stimuli were applied during the peak of the theta frequency band. Similarly, when stimuli were locked to the trough of the theta rhythm, induction of homosynaptic LTD was obtained (Huerta and Lisman, 1995). These observations demonstrate the potential capability of phase-dependent stimulation to modify synaptic strength in both directions, and facilitate synchronization or desynchronization of the neuronal population (Martin et al., 2000; Kauer and Malenka, 2007; Pfister and Tass, 2010).

In another animal study conducted by Siegle and Wilson (2014), the dorsal CA1 was stimulated in freely behaving mice at specific phases (peak or trough) of theta band activity. The mice were trained to perform a spatial memory task including encoding and decoding stages. During the encoding stage memory performance was improved as result of stimulation near the peak of the cycle. However, delivering stimulation at the trough of the theta cycle during the retrieval stage enhanced performance for retrieval (Siegle and Wilson, 2014). The results of this study demonstrate the possibility of altering behavior by theta rhythmic phase-dependent stimulation. Since there is a direct correlation between CFC and performance (Tort et al., 2008), there is a strong possibility that phase-dependent stimulation could potentially modulate the level of CFC and consequently alter behavior as well.

Despite the growing interest in phase-dependent stimulation, the number of studies regarding stimulation at specific phases of brain oscillations such as the theta or beta bands in human subjects is very limited likely due to technical challenges (Chen et al., 2011, 2013). However, recent studies show improvements in the efficacy of DBS in severe essential tremor during stimulation of the thalamus while recording tremor amplitude and phase and adjusting the timing of the stimulation to one half of the tremor cycle (Cagnan et al., 2013, 2015). As another supportive example, a recent study demonstrated phase-dependent plasticity effects in the motor cortex in awake humans using transcranial magnetic stimulation (TMS). The TMS pulses were phase-locked to the sensorimotor mu rhythms and synchronized with the trough (more excitable) or peak (less excitable). The results successfully showed an increase in motor cortical excitability similar to the modulation of the LTP effects during more excitable phases (trough) and no changes in less excitable phases (peak) (Zrenner et al., 2017).

## Illustrative Cases

Previously, we measured ECoG signals in the gamma frequency band in epilepsy subjects performing a recall task for sequences of images over a certain timespan. We provided evidence suggesting that the recall phase was characterized by a marked increase of power in the gamma frequency band, but once the sequences were learned, gamma band activity decreased (Madhaven et al., 2015). Gamma band activity decreased in magnitude as subjects participated in more trials and made improvements in visual memory learning. In this initial illustrative study, we designed a similar task, consisting of multiple trials, but with a focus on CFC, specifically the coupling between the phase of theta and the amplitude of the gamma band.

### Human Subjects

Behavioral and electrophysiological data were collected from a patient with drug-resistant epilepsy undergoing invasive monitoring as a typical part of the clinical protocol for identifying seizure focus, who was subjected to monitoring for potential surgical resection. The second participant was a patient with Parkin son’s disease undergoing DBS lead placement surgery. The Institutional Review Board at the Johns Hopkins School of Medicine approved all steps in the research project and informed consent was obtained from the subjects.

### ECoG Recording Protocol

For the epilepsy patient, the clinical team surgically implanted multiple intracranial subdural electrodes, including a grid and strips, with 2.3 mm diameter recording contacts (1 cm separation, Ad-Tech, Racine, WI, United States), directly on the surface of the cortex. Ictal onset localization was performed to guide any subsequent surgical resection. In accordance with the clinical procedure for identifying seizure foci, the recording elements were also placed over brain regions outside and neighboring the anticipated seizure focus area. After undergoing a craniotomy for subdural implantation of electrodes, the subject underwent 1 week of close monitoring and clinical observation. The electrode location map was estimated by co-registering the preoperative magnetic resonance imaging (MRI) with the post-operative computed tomography (CT) scans. A 3-dimensional brain surface was reconstructed, followed by parcellation using Freesurfer (Destrieux et al., 2010). The electrode positions were mapped onto discrete brain areas (Destrieux et al., 2010). Based on these coordinates, each electrode contact was superimposed on the reconstructed brain surface for illustrating the electrode coverage and, for verifying the location of the task responsive channels.

For the Parkinson’s disease subject, a subdural ECoG strip electrode was placed during DBS lead placement surgery. A preoperative MRI study was used to plan the cortical target. A point on the primary motor cortex (M1) was identified, approximately 3 cm from the midline, based on the anatomical identification of the central sulcus. The primary goal was to target the hand and arm representation area of M1, slightly medial to the ‘hand knob’, in a similar parasagittal plane as the typical entry site for placement of the subthalamic nucleus DBS electrodes. After drilling the left frontal burr hole and opening the dura mater, an eight-contact subdural ECoG strip (2.3 mm contacts, 1 cm spacing) was placed on the brain surface and directed posteriorly in a parasagittal plane to increase the probability of contacts covering M1 and the primary sensory cortex (S1). The location of the burr hole was finalized by the selection of the safest entry point for the intended DBS lead placement trajectory with no additional skull or scalp exposure needed for subdural strip placement. The burr hole was sealed with a fibrin sealant after placement of the subdural strip (and guide tube for microelectrode recording and DBS lead placement) (Crowell et al., 2012).

### Visual Memory Task

**FIGURE 1:**
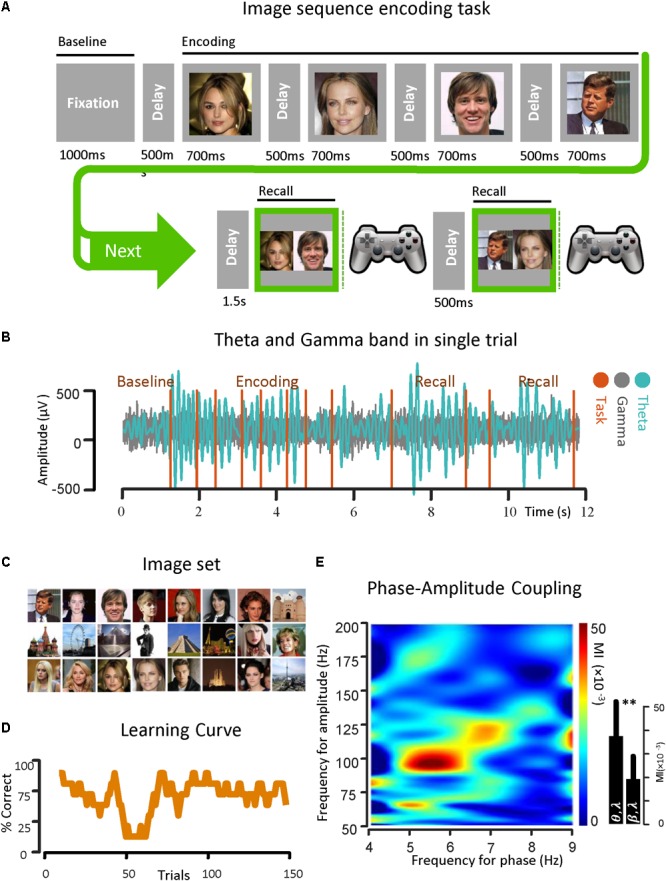
Theta band phase and gamma band amplitude coupling in an epilepsy subject. **(A)** An image sequence encoding task is used for the investigation of PAC in the temporal lobe. The ECoG signal was recorded from strip and depth electrodes implanted throughout the temporal and frontal lobes, while the subject learned the temporal-order of multiple sequences of images over trials through repeated recall. Here the temporal sequence of the task is shown. **(B)** A sample of ECoG signal recorded from the parahippocampal gyrus of a subject with epilepsy in both theta band (13–30 Hz) and gamma band (50–200 Hz) is shown. The vertical task lines correspond to different phases of the behavioral task. **(C)** The image set that is used in the task. **(D)** Behavioral performance of the subject is shown. Here, the average performance by considering the percentage of correct trials (*y*-axis) with respect to the trial number in bins of 10 trials (*x*-axis) is illustrated. **(E)** Examples of phase–amplitude coupling observed in the parahippocampal gyrus of the same subject during recall phases of the task. Modulation indices over a wide range of frequencies for phase and amplitude show a strong coupling between the phase of the theta-rhythm (4–9 Hz) and the amplitude of gamma-activity (50–200 Hz). ^∗∗^Means significant change (two-sample *t*-test, *p* = 0.01).

The epilepsy subject participated in a four-image version of a sequence recall task. In this task, the subject was instructed to learn and later recall the order of four-image sequences through repeated recall. Instructions for the task included information that multiple image sequences would be presented during the session, and memory for sequence order would be tested on every trial following a delay period after sequence presentation (Figure 1A). The subject was also informed that each individual sequence would be repeated multiple times to facilitate learning. There was no encouragement to say the sequence aloud or talk during the task so as to avoid speech artifacts. To probe visual memory learning, stimuli took the form of celebrity faces and various well-known buildings. Stimuli were selected randomly from a set of images (for example, from a set of 24 images for 4-image sequence) presented on a 15 inch (screen diagonal: 15.40 inch, width: 13.06 inch, height: 8.16 inch) Macbook Pro laptop computer, using the MATLAB Psychophysics toolbox Figure 1A,B (Brainard, 1997). Electrode recordings were correlated with behavioral events via digital pulses (Madhaven et al., 2015).

Before the onset of a new image sequence, a 500 ms time fixation interval was used to establish baseline recordings for each subject as they performed the task. The interval during which subjects were presented with a sequence is referred to as the Encoding Phase. Images within a given sequence were always presented in the same order. Sequences were displayed in pseudo random order with each image in the sequence being projected onscreen for 700 ms and immediately followed by a gray screen for 500 ms. Following a delay of 1.5 s after sequence termination, a choice screen was presented, initiating the Recall Phase. The choice screen consisted of a side-by-side comparison of a randomly chosen pair of images from the sequence. The subject was then asked to indicate which image had appeared earlier in the sequence using one of two buttons on a Logitech gamepad controller. The subject was given another choice screen 500 ms after the first, with each choice screen in the Recall Phase constituting a separate trial in the analysis. Size and quality of the images on the choice screen were the same as during the sequence presentation, and the position of the individual images on the choice screen (left or right) was randomized in each trial. Testing continued until the subject met any of the stopping criteria, as defined by Madhaven et al. (2015).

For this protocol, testing was terminated if the subjects were able to give 8 consecutively correct recalls within a minimum of 20 trials. We calculated power across various frequency bands, along the three main task phases: (1) Baseline Phase: [0, 500 ms] before image presentation, (2) Encoding Phase: [0, 700 ms] from image onset for each image in the sequence, and (3) Recall Phase: [0, key press time] from the presentation of the first choice screen. There were two choice screens for each Recall Phase. All analyses were based on the initial 500 ms after onset of each choice screen to capture the physiological responses correlated with recall before onset of the behavioral response. All of the task phases recorded were 500 ms in duration for consistent comparisons across epochs (Madhaven et al., 2015).

### Center-Out Reaching Task

For the recorded PD subject undergoing DBS lead placement surgery, a center-out reaching task with eight targets was implemented. The patient in the OR was asked to move a cursor with his/her finger on the touch screen monitor from the center toward eight different targets located in the periphery. The cursor diameter is 1 cm and may be moved by the subject’s finger to the same size target up to 10 cm away. A prompting cursor at the center of the screen is first shown. After the subject touches the cursor, a target appears and the subject is asked to prepare the movement. This state is called the HOLD state and has the highest level of the PAC with respect to the rest of the task (De Hemptinne et al., 2013). The subject initiates hand motion when the cursor color changes (which is considered the GO signal). The trajectory of the finger on the touchscreen monitor is shown during the hand movement. When the subject’s hand reaches the target the color changes again. This constitutes a successful trial and the next trial starts after a short delay (between 1 and 2 s). The baseline center–out reaching task was composed of 100 trials. The paradigm was kept as simple as possible but the number of targets and the number of trials could be increased with respect to the complexity of task.

### Spectral Analyses

For both illustrative cases, we analyzed ECoG signals (at 1 kHz sampling frequency) using band pass filtering in conjunction with the Hilbert transform. We first filtered the raw signal in each frequency band (theta, beta, or gamma band) using a second-order Butterworth band pass filter in both the forward and reverse directions with zero phase distortion. Next, we applied the Hilbert transform, which yields a complex number. We took the absolute value (analytic amplitude) of the Hilbert transform to extract the instantaneous power of the signal (Madhaven et al., 2015).

### Modulation Index

To quantify the amplitude modulation by phase, a modulation index (MI) is defined based on a normalized entropy measure (Tort et al., 2010). Let *f_A_* and *f_P_* denote the amplitude and the phase frequency ranges of interest, respectively, and *S(t)* denote ECoG signal in the specific time interval. The MI is estimated by the following process:

(I) S(t) is filtered within the f_A_ and f_P_ bandpasses. The filtered signals are denoted as Sf_P_(t) and Sf_A_(t).
(II) The phase of Sf_P_(t) [denoted as ϕ_fp_(t)] and amplitude envelope of Sf_A_(t) [denoted as A_fA_(t)] are obtained from the standard Hilbert transform of xf_p_(t) and xf_A_(t), respectively. The two dimensional signal [ϕ_fp_(t), A_fA_(t)] is then generated, which includes the amplitude of the f_A_ at each phase of the f_P_.
(III) *The phases ϕ_fp_(t) are binned into N intervals, and the mean of A_fA_ over each phase bin is calculated [denoted as*< A_fA_ > _φ_fP__(j)*].*
(IV) *The mean amplitude*< A_fA_ > _φ_fP__(j) *is normalized by dividing each bin value by the sum over the bins (N is the number of bins).*

$$\begin{array}{c} P_{j}=\frac{{<A}_{\mathrm{fA}}{>}_{\varphi_{\mathrm{fP}}}\left(j\right)}{{\sum_{j=1}^{N}{<A}_{\mathrm{fA}}{>}_{\varphi_{\mathrm{fP}}}\left(j\right)}_{}^{}} \end{array}$$

(V) Since the normalized P_j_ values have the discrete probability density function characteristics, the modulation index could be defined by normalizing the Kullback–Leibler (KL) distance (Kullback and Leibler, 1951) of the observed amplitude distribution (P) from the uniform distribution (U) to the maximum possible entropy value (log N) (Tort et al., 2010).

$$\begin{array}{c} MI=\frac{D_{\mathrm{KL}}\left(P,U\right)}{\mathrm{Log}\;\left(N\right)} \end{array}$$

Small MI values indicate low phase-amplitude modulation, while larger MI values result from high phase-amplitude modulation. The two-dimensional plot is obtained by estimating the MI values in the amplitude and the phase frequency ranges of interest (Tort et al., 2008, 2009; Scheffer-Teixeira et al., 2012).

### Phase–Dependent Stimulation

We created a technique for phase-dependent stimulation based on recorded rhythmic activity present in ECoG signals recorded from the motor cortex (in a Parkinson’s disease subject) and the parahippocampal gyrus (in an epilepsy subject). Here, we did not apply actual stimulation pulses experimentally and only demonstrated the performance of the phase–dependent stimulation timing algorithm utilizing the recorded signals to illustrate the feasibility of this technique. For the former case the phase of the beta band is estimated and for the latter the phase information in the theta band is of interest. In order to estimate the instantaneous frequency and phase of the ECoG signal at a specific point in time with the necessary accuracy and precision an autoregressive (AR) modeling approach is used. The AR model enables us to predict the ECoG signal phase and frequency and deliver phase-dependent stimulation pulses with low timing latency. The algorithm is comprised of several sequential blocks including frequency band optimization within a predefined frequency band, autoregressive spectral estimation, estimating the future signal by autoregressive time-series prediction, calculating the instantaneous frequency and phase via the Hilbert-transform analytic signal, and calculating the time lag until the desired phase for the output stimulation pulse The parameters to be optimized include the frequency band optimization bandwidth, the AR order, the time window for time-series forward prediction, and the band pass filter order and type. These parameters are estimated for each dataset presented in this study separately based on baseline recording session of each experiment in an offline fashion (Chen et al., 2011, 2013).

### Statistical Analyses

For each data set, the level of coupling (estimated by MI) between the phase of the beta-rhythm (13–30 Hz) and the amplitude of gamma-activity (50–300 Hz), is compared with the theta band (4–9 Hz) and gamma band (50–200 Hz) coupling. The mean of the MIs are compared using a two-sample *t*-test (*p* < 0.05 was considered significant) to assess for statistical significance.

## Potential Technique for CFC Modulation

In general, CFC refers to a statistical relationship between instant frequency, phase, and amplitude in two different frequency bands, and it has been quantified in both animal studies and human subjects. It also has been recorded in many brain regions, and associated with several cognitive processes and disease states (Canolty and Knight, 2010). CFC analyses require both a high signal to noise ratio and a high temporal resolution of electrophysiological measurements. Therefore, ECoG signals are useful for exploring different properties of this phenomena. Here, we used ECoG signals recorded from the motor cortex of a Parkinson’s disease patient and the temporal lobe of a patient with epilepsy to illustrate the beta–gamma and theta–gamma, PAC, respectively. Additionally, we applied the phase-dependent stimulation algorithm to both cases to demonstrate a potential technique for modulation of the CFC level.

### Theta–Gamma Coupling Illustration

For demonstrating the coupling between the phase of the theta rhythm and the amplitude of the gamma oscillation, an image sequence learning task was used with an epilepsy subject for investigating PAC in the temporal lobe. The ECoG signals were recorded from the strip and depth electrodes implanted in or on temporal and frontal cortical areas. During the data recording session, the subject performed a temporal-order learning task, consisting of multiple sequences of images over trials through repeated recall. In Figure 1A, the temporal sequence of the task is shown. A sample of ECoG signal recorded from the parahippocampal gyrus of the subject in both theta band (4–9 Hz) and gamma band (50–200 Hz) is also shown (Figure 1B) with respect to time. In Figure 1C, the phase–amplitude coupling estimation in the parahippocampal gyrus of the same subject is illustrated. We used the Recall Phase of the task since it has a strong correlation with the behavioral performance (Tort et al., 2009; Axmacher et al., 2010). Modulation indices over a wide range of frequencies for the phase of low frequency oscillation (0–50 Hz) and the amplitude of the rhythmic activities in the high frequency band (50–350 Hz) for 5 s in two recall phases for one session (*n* = 50, trials), detected a stronger coupling between the phase of the theta rhythm and the amplitude of gamma activity (MI = 0.038 ± 0.0154) compared to the phase amplitude coupling in the beta–gamma range (MI = 0.0182 ± 0.011).

### Beta–Gamma Coupling Illustration

**FIGURE 2:**
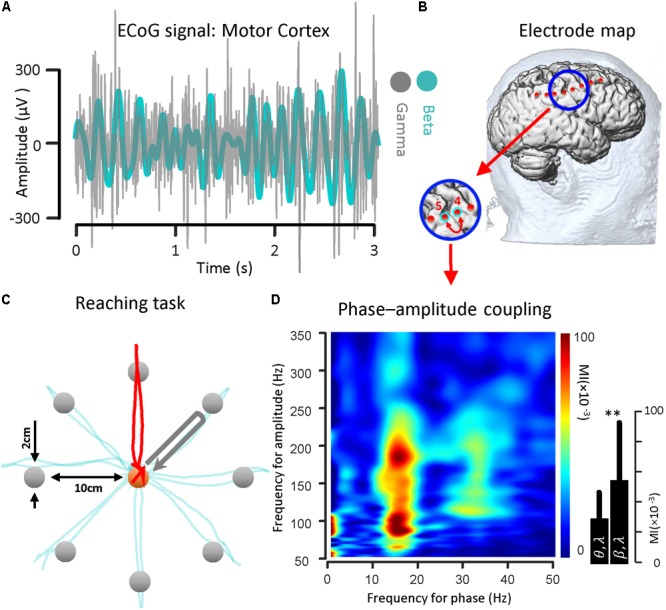
Beta band phase and gamma band amplitude coupling in a PD subject. **(A)** A sample of ECoG signal recorded from primary motor cortex (contact number 4, during the HOLD phase of the reaching task) of a subject with Parkinson’s disease in both beta band (13–30 Hz) and gamma band (50–200 Hz) is shown. **(B)** The electrode coordinates of the implanted cortical strip are estimated using preoperative MRI, intraoperative fluoroscopy and intraoperative CT. The relative location of the strip electrode contacts with respect to the site of recording is shown. **(C)** The ECoG signals were recorded while the subject performed a center-out reaching task. In this task, eight targets were distributed equally on a peripheral circle, and the patient in the OR moved a cursor with a finger on the touch screen monitor from the center toward four different marked target locations. The ECoG signal used as a sample was recorded during HOLD phase of task while the subject performed the reaching task toward the 90 degree target (highlighted with a red trajectory). **(D)** Examples of phase–amplitude coupling observed in the motor cortex of the PD patient. Modulation indices over a wide range of frequencies for phase and amplitude show a strong coupling between the phase of the beta-rhythm and the amplitude of gamma-activity (50–300 Hz). ^∗∗^Means significant change (two-sample *t*-test, *p* = 0.01).

To illustrate the coupling between the phase of the beta rhythm and the amplitude of the gamma oscillation, we used ECoG signals recorded from the motor cortex via strip electrode contacts implanted temporarily during DBS lead placement surgery. The implanted cortical strip electrode coordinates were estimated using preoperative MRI, intraoperative fluoroscopy and postoperative CT. In Figure 2B, the relative location of the strip electrode contacts with respect to the site of recording is shown. A sample of ECoG signal recorded from primary motor cortex (contact number 4 in Figure 2B) during the HOLD state (waiting for the GO signal) in both beta band (13–30 Hz) and gamma band (50–350 Hz) is illustrated (Figure 2A). The gamma band activity bandpass was held wide to detect a broad range of PAC signal. The ECoG signals were recorded while the subject performed a center-out reaching task (the intended target and trajectory of the hand are shown in Figure 2C). In this task, eight targets were distributed equally on a circle perimeter and the patient in the operating room moved a cursor with his finger on the touch screen monitor from the center toward the eight different targets located in the periphery (Figure 2C). For demonstrating the phase–amplitude coupling in the motor cortex of the PD patient, the modulation indices over a wide range of frequencies were estimated during the HOLD state of the task while waiting for the GO signal which demonstrated the highest level of PAC (De Hemptinne et al., 2013). Figure 2D illustrates the MI level of the phase of the low frequency range (13–30 Hz) coupled to the amplitude of the high frequency oscillation (50–350 Hz) using a 10 s recording length. The figure demonstrates a stronger coupling between the phase of the beta-rhythm (13–30 Hz) and the amplitude of gamma-activity (50–300 Hz), (MI = 0.056 ± 0.0382) in comparison to the theta band (4–9 Hz) and gamma band (50–200 Hz) coupling (MI = 0.030 ± 0.0186) for one block (*n* = 50, trials) during the HOLD phase of the task.

### Phase-Dependent Stimulation for CFC Modulation

**FIGURE 3:**
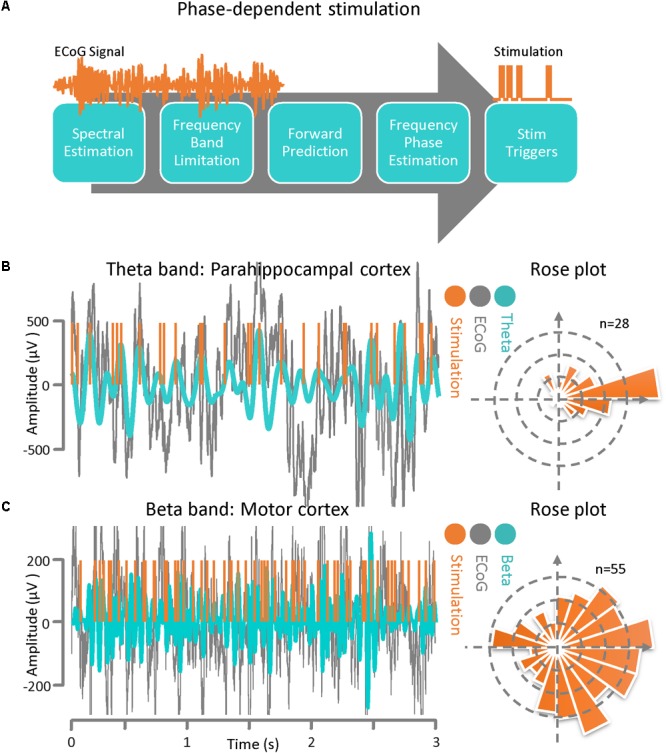
Phase–dependent stimulation. **(A)** A general block diagram and the sequence of phase dependent stimulation steps is demonstrated. The main goal of this algorithm is to be able to deliver phase-dependent stimulation pulses in real-time by estimating the instantaneous phase and frequency of the ECoG signal at a specific time point. The algorithm is comprised of several sequential blocks including frequency band optimization, autoregressive spectral estimation, estimating the future signal by autoregressive time-series prediction, calculating the instantaneous frequency and phase via the Hilbert-transform analytic signal, and calculating the time lag until the desired phase for the output stimulation pulse. **(B)** ECoG signal from an epilepsy patient with theta-band activity. Results of the phase-dependent stimulation algorithm at the peak of the theta rhythm and a Rose plot of phases at which stimulation occurred. **(C)** ECoG signal from a Parkinson’s disease patient demonstrating beta-band activity. Results of the phase-dependent stimulation algorithm at the peak of the beta rhythm and a Rose plot of phases at which stimulation occurred.

The phase-dependent stimulation technique is a relatively new neuromodulation approach for delivering electrical stimulation to a target cortical region at a specific phase of the detected rhythmic activity. This technique may be realized via an autoregressive signal modeling based-approach (Chen et al., 2011, 2013). The main goal of this algorithm is to be able to deliver phase-dependent stimulation pulses in real time by estimating the instantaneous phase and frequency of the ECoG signal at a specific time. In Figure 3A, a general block diagram and sequence for phase-dependent stimulation is demonstrated. The algorithm is comprised of several sequential tasks including frequency band optimization within a predefined frequency range, autoregressive spectral estimation, estimation of the future signal by autoregressive time-series prediction, calculating the instantaneous frequency and phase via the Hilbert-transform analytic signal, and calculating the time lag until the desired phase for the output stimulation pulse. Prior to active stimulation at a specified phase on a selected channel, the AR modeling parameters were optimized based on Akaike’s Information Criterion (Akaike, 1974) (either online or offline) with respect to the application. We set the model parameters as: AR order = 22, λ = 0.69, Filter order = 2, Filter type = Butterworth, and t_0_-t_stop_ = 150 ms for the Parkinson’s disease patient and we used AR order = 20, λ = 0.75, Filter order = 2, Filter type = Chebyshev, and t_0_-t_stop_ = 110 ms for the epilepsy patient (Chen et al., 2011, 2013). For demonstrating the method’s performance of phase-dependent stimulation on theta band oscillations, the ECoG signal was recorded from the parahippocampal gyrus (Figure 1). The results of the phase-dependent stimulation trigger timing at the peak of the theta rhythm and the Rose plot of phases at which stimulation would have occurred is shown in Figure 3B. Additionally, the ECoG signal collected from the motor cortex of a Parkinson’s disease patient was used to demonstrate the performance of the phase-dependent stimulation algorithm in the beta band. Results of the timing for stimulation at the peak of the beta rhythm and the Rose plot of phases at which stimulation would have occurred are also demonstrated (Figure 3C). The phase-dependent stimulation trigger times in Figure 3B,C are in offline mode and no active stimulation is involved.

## Concluding Remarks

Despite the potential relevance of CFC for understanding normal or pathological brain function, current CFC analysis has many limitations. The definition of instantaneous phase and amplitude and their estimation in a limited frequency band, the non-stationary nature of the ECoG signals exhibiting spectral correlations between frequency components, and the use of surrogate data to estimate the significance of the estimated CFC, are sometimes used to argue that the detected interactions between neural frequency components are mostly due to common non-stationarities in the signals (Aru et al., 2015). In addition to computational constraints, which are mostly at the software level, there are other limitations at the hardware level as well specifically in real-time applications which are dependent on high spatial and temporal resolution in the recorded signals used in conventional CFC detection methods. However, even with limitations and several methodological challenges, CFC is still earnestly researched, in both basic and translational neuroscience including the neurobiology of central nervous system diseases.

Based on studies that are briefly reviewed here, CFC may appear in a variety of forms and emerges from different neuronal architectures representing distinct behaviors or associations with neurological disorders. However, the causal relationship between neural CFC and brain function or diseases needs to be demonstrated. One of the major reasons for the sparcity of causal investigations, is the lack of CFC modulation techniques. If we consider cortical oscillations as rhythmic modulations of neuronal excitability (Fries, 2005), then the neuronal rhythms should modulate local processing with respect to the relative timing of the stimulus to the phase of the low frequency oscillations which reflected in CFC measures. Phase-dependent stimulation might be a potential solution for CFC modulation and more importantly, might provide a framework for causality assessment. For instance, if local cortical excitability is associated with the peak of low frequency rhythms, then stimuli time-locked to the peak of the oscillation might be processed more efficiently than other phases, and increasing the level of CFC.

In this hypothesis and theory article, we have attempted to summarize evidence demonstrating the function of CFC as a key mechanism for the coordination of neural dynamics across spatial scales and temporal domains. CFC markers have been observed and related to local and network processing in learning and memory related tasks both in humans and animals. Additionally, CFC has also been employed to investigate neurological psychiatric disorders such as schizophrenia, epilepsy, Alzheimer’s disease, and Parkinson’s disease. We discussed Parkinson’s disease more in detail and reviewed CFC abnormalities related to disease states. Importantly, we also described that excess levels of CFC return to normal after effective therapeutic treatments such as dopaminergic medication and DBS. This is a preliminary step toward using CFC as a biomarker for monitoring the state of the disease and evaluating therapeutic efficacy in PD patients.

In conclusion, we described the existing evidence on the potential capability of phase-dependent stimulation for modulating CFC. This may be useful for selectively altering brain function or improving disease states. In two different illustrative cases, we analyzed the CFC of the ECoG signals recorded from the motor cortex of a Parkinson’s disease patient and the parahippocampal gyrus of an epilepsy patient and demonstrated the performance of a real time phase detection and phase specific stimulation algorithm without actual stimulation. Although, the presented technique has potential application for the modulation of CFC measures, many technological issues such as real time ECoG recording and processing and stimulation artifacts interfering with the recordings would remain as challenges for this type of neuromodulation, Finally, phase-dependent stimulation in specific frequency ranges requires low latency access to the ECoG signals. It also needs an optimal design of the processing blocks with the fastest possible implementation techniques. We mentioned some of the existing challenges for detecting, processing, and possible modulation of CFC using phase-dependent stimulation techniques, and emphasized the need for developing state of the art technologies to build upon CFC based neuromodulation devices in the future.

## Author Contributions

YS and WA performed the experiments, analyzed the data, and wrote the manuscript. WA and YS designed the experiments. WA performed the neurosurgical procedures and electrode implantations. YS ran the experiments and collected the data. YS and WA commented and approved the manuscript.

## Conflict of Interest Statement

WA serves on the Advisory Board of Longeviti, LLC, and acts as a consultant for Globus Medical. The remaining author declares that the research was conducted in the absence of any commercial or financial relationships that could be construed as a potential conflict of interest.
